# Comparison of population-averaged and cluster-specific models for the analysis of cluster randomized trials with missing binary outcomes: a simulation study

**DOI:** 10.1186/1471-2288-13-9

**Published:** 2013-01-23

**Authors:** Jinhui Ma, Parminder Raina, Joseph Beyene, Lehana Thabane

**Affiliations:** 1 Department of Clinical Epidemiology and Biostatistics, McMaster University, Hamilton, Ontario, Canada; 2 McMaster University Evidence-based Practice Center, Hamilton, Ontario, Canada; 3 Biostatistics Unit/FSORC 3rd Floor Martha, Room H325, St. Joseph's Healthcare Hamilton, 50 Charlton Avenue East, Hamilton, Ontario, L8N 4A6, Canada; 4 Centre for Evaluation of Medicines, St Joseph’s Healthcare, Hamilton, Ontario, Canada; 5 Population Health Research Institute, Hamilton Health Sciences, Hamilton, Ontario, Canada

**Keywords:** Marginal model, Population-averaged model, Cluster-specific model, Multiple imputation, Cluster randomized trial, Covariate dependent missingness, Generalized estimating equations, Random-effects logistic regression

## Abstract

**Abstracts:**

**Background**

The objective of this simulation study is to compare the accuracy and efficiency of population-averaged (i.e. generalized estimating equations (GEE)) and cluster-specific (i.e. random-effects logistic regression (RELR)) models for analyzing data from cluster randomized trials (CRTs) with missing binary responses.

**Methods**

In this simulation study, clustered responses were generated from a beta-binomial distribution. The number of clusters per trial arm, the number of subjects per cluster, intra-cluster correlation coefficient, and the percentage of missing data were allowed to vary. Under the assumption of covariate dependent missingness, missing outcomes were handled by complete case analysis, standard multiple imputation (MI) and within-cluster MI strategies. Data were analyzed using GEE and RELR. Performance of the methods was assessed using standardized bias, empirical standard error, root mean squared error (RMSE), and coverage probability.

**Results**

GEE performs well on all four measures — provided the downward bias of the standard error (when the number of clusters per arm is small) is adjusted appropriately — under the following scenarios: complete case analysis for CRTs with a small amount of missing data; standard MI for CRTs with variance inflation factor (VIF) <3; within-cluster MI for CRTs with VIF≥3 and cluster size>50. RELR performs well only when a small amount of data was missing, and complete case analysis was applied.

**Conclusion**

GEE performs well as long as appropriate missing data strategies are adopted based on the design of CRTs and the percentage of missing data. In contrast, RELR does not perform well when either standard or within-cluster MI strategy is applied prior to the analysis.

## Background

Cluster randomized trials (CRTs) are randomized controlled trials in which clusters of subjects rather than independent subjects are randomly allocated to trial arms and outcomes are measured for individual subjects or clusters. CRTs increasingly are being used in health services research and primary care. Reasons for adopting cluster randomization as a more appropriate design include: 1) administrative convenience; 2) ethical considerations; 3) intervention is naturally applied at the cluster level; 4) to enhance the subject compliance; and 5) to minimize the potential treatment “contamination” between the intervention and control subjects [1]. In CRTs, outcomes from subjects within the same cluster may exhibit a greater correlation than do outcomes from subjects in different clusters. The correlation within clusters, which is quantified by the intracluster correlation coefficient (ICC) ρ, may result in substantially reduced statistical efficiency relative to trials that randomize the same number of individuals. The overall outcome variance *σ*^2^ in a CRT can be expressed as the sum of between-cluster variance *σ*_*B*_^2^ and within-cluster variance *σ*_*W*_^2^. Correspondingly, the ICC is defined as *ρ* = *σ*_*B*_^2^/(*σ*_*B*_^2^ + *σ*_*W*_^2^), which is interpreted as the amount of variation that can be explained by variation between clusters. The reduction in efficiency is a function of the variance inflation due to clustering, also known as the design effect or variance inflation factor (VIF), given by $\text{VIF}=1+\left(\bar{m}-1\right)\rho$, where $\bar{m}$ denotes the average cluster size.

Missing data may be a serious problem in some CRTs due to the lack of direct contact with individual subjects and lengthy follow-up [2]. The impact of missing data on estimation of the treatment effect and its confidence interval depends on the mechanism which caused the data to be missing, the strategy used to handle missing data, and the model used for statistical analysis. Missing data mechanisms are generally categorized into missing completely at random, missing at random, and missing not at random [3]. An observation is missing completely at random if the probability of missingness is independent of any observed or unobserved measurements. An observation is missing at random if the probability of missingness depends on the observed data. Missing not at random is the situation where the probability of missingness is related to the unobserved measurement. Covariate dependent missingness (CDM) is a simple case of missing at random, where the probability of missingness depends only on the observed covariates, but not on the observed outcomes. In this study, we focus on missing outcome data since the chance of having missing data on baseline characteristics or covariates, which are typically collected prior to the randomization, is relatively low. A variety of methods can be used to handle missing data: 1) listwise deletion (or complete case analysis) which excludes subjects with missing data from the analysis; 2) single imputation procedures, such as mean imputation; 3) likelihood-based methods, which usually involve a maximization of the likelihood function derived from the underlying model and estimate parameters by accounting for the missingness; and 4) multiple imputation (MI), which replaces each missing value with a set of plausible values that represent the uncertainty about the right value to impute. MI has become widely-used methods for handling missing data in recent years since it offers distinct advantages over other methods. Single imputation methods do not account for the uncertainty about the predictions of the unknown missing values and may lead to underestimation of the variance of effect estimates. Likelihood-based methods may be difficult to implement when no algorithm or procedure is available to maximize the likelihood. Listwise deletion weakens the statistical power of the test conducted and may lead to biased results when data are missing not at random. In addition, this analysis does not adopt an intention-to-treat principle — because it excludes some randomized participants (i.e. those with missing data). In general, compared to complete case analysis and single imputation strategies, MI has the advantage of reducing the bias and improving the efficiency of effect estimates, even when only outcome data are missing [4,5]. Moreover, for cluster randomized trials, there is a higher risk that the variance of effect estimate will be underestimated with single imputation methods. MI would be, therefore, the most appropriate choice to handle missing outcome data to minimize the likelihood of underestimating the variance. Our previous study showed that MI, if used appropriately, is a valid strategy to handle missing binary outcomes in CRTs [6].

A key property of CRTs is that inferences or analyses are frequently done to apply at the individual level while randomization is at the cluster level, thus the unit of randomization may be different from the unit of inference or analysis. In this case, the lack of independence among individuals in the same cluster, i.e. the between-cluster variation, presents special methodological challenges that affect both the design and analysis of CRTs. Consequently, standard approaches for statistical analysis do not apply because they may result in severely underpowered studies and spuriously elevated Type I error rates [1]. Different statistical methods that account for the clustering effect have been proposed in the literature, and they are categorized into individual-level and cluster-level data analysis methods. Individual-level analysis models, such as population-averaged (PA) models (also called marginal models) and cluster-specific (CS) models (also called conditional models), have been advocated for the analysis of CRTs with binary outcomes since they allow for the possible imbalance of both cluster-level and individual-level characteristics to be incorporated into the analysis. The ability to adjust for imbalanced characteristics between trial arms is very important when the number of clusters is not large enough to keep the cluster- or individual-level characteristics balanced between the trial arms. The generalized estimating equations (GEE) approach [7] and the random-effects logistic regression (RELR) are two commonly used individual-level analysis methods for estimating the PA and CS intervention effect for CRTs with binary outcomes, respectively.

Some attention has been paid in the literature to the performance of GEE approach and RELR in the analysis of binary outcomes in CRTs. Austin [8] compared their statistical powers through a simulation study in which the minimum number of clusters examined was 26 (13 clusters per trial arm). The results showed that the differences between the two methods were negligible in most settings. Bellamy *et al.*[9] also conducted a series of simulation studies comparing their statistical power. They examined settings in which the total number of clusters was 10, 20, 30 or 50, the mean number of subjects per cluster was either 10 or 100, the ICC was 0.1, the response proportion in the control arm was 0.23 and the response proportions in the intervention arm were: 0.09, 0.13, 0.18, 0.23 or 0.28. The study showed that the difference between the two models diminished as the number of clusters increased. In particular, the difference was negligible if the total number of clusters was at least 30. However, if the total number of clusters was 10 or 20, RELR would have moderately lower power than GEE method. Ukoumunne *et al.*[10] compared the accuracy of estimated treatment effect and confidence interval coverage of several methods for analyzing binary outcomes in CRTs through a simulation study. They showed that the GEE method had acceptable properties as long as the bias of the standard error was corrected when the number of clusters was small. The RELR was not assessed in their simulation study. Ma *et al.*[11] compared different strategies to handle missing binary outcomes in CRTs when GEE method and RELR were used as methods of analysis. Findings from this paper implied that both models could be used to analyze data from CRTs after multiple imputation was applied to handle missing outcome data. However, the generalizability of their findings was limited in that their simulation was based on a real dataset. This study is an extension of this work to assess the accuracy and efficiency of PA and CS models, in particular, the GEE method and the RELR respectively, when multiple imputation techniques are applied to handle missing binary outcomes in CRTs using simulated data. The performance of the methods is compared in terms of standardized bias, empirical standard error, root mean squared error (RMSE), and coverage probability. The simulation is designed under the assumptions of CDM and CRTs with a balanced completely randomized design.

## Methods

The rest of this section is organized as follows: First, the statistical analysis methods (i.e. GEE and RELR) used to analyze binary outcomes in CRTs are described. Second, the missing data strategies used in this study for handling missing binary outcomes are briefly introduced. Third, the method for combining the results across multiply imputed datasets is described.

### Statistical analysis methods

#### Generalized estimating equations

The GEE approach for fitting the logistic regression developed by Liang and Zeger [12] can be formulated as

(1) $$\text{logit}(\text{Pr}\left(y_{\text{ijl}}=1\right))=X_{\text{ijl}}\beta_{\text{marginal}}\text{,}$$

where *y*_*ijl*_ denotes the binary outcome of patient *l* in cluster *j* in the intervention group *i*, Pr(*y*_*ijl*_ = 1) denotes the corresponding probability of success, *X*_*ijl*_ denotes the corresponding vector of individual-level or cluster level covariates. *β*_*m* arg *inal*_ denotes the marginal regression coefficients, and $\text{logit}(\text{Pr}\left(y_{\text{ijl}}=1)\right)=\text{log}\left(\frac{\text{Pr}\left(y_{\text{ijl}}=1\right)}{1-\text{Pr}\left(y_{\text{ijl}}=1\right)}\right)$.

To analyze the data from CRTs, an exchangeable correlation matrix is usually specified to account for potential within-cluster homogeneity in outcomes, and the robust standard error method is used to obtain the improved standard error for estimation of *β*_marginal_. In this paper, we only include one covariate, treatment group, in the model fitting.

It has been recommended that at least 40 clusters need to be included in a study to ensure the GEE method produces reliable standard errors [13]. This is because, firstly, the method tends to underestimate the covariance of observations leading to downward biased estimate of standard error and, secondly, the estimate of standard error is highly variable when the number of clusters is too small [14]. A number of methods have been proposed for dealing with the shortcomings of the robust standard error estimator [13]. In this paper, the downward bias of the sandwich standard error estimator is adjusted by multiplying it by $\sqrt{J/\left(J-1\right)}$, where J is the number of clusters in each arm.

#### Random-effects logistic regression

RELR incorporates cluster-specific random effects into the logistic regression and assumes that the random effects follow a normal distribution. The model can be formulated as

(2) $$\text{logit}(\text{Pr}\left(y_{\text{ijl}}=1\right))=X_{\text{ijl}}\beta_{\text{conditional}}+U_{ij}\text{,}$$

where *U*_*ij*_ ~ *N*(0, *σ*_*B*_^2^) represent the random effects, which vary independently from one cluster to another according to a common Normal distribution with a mean of zero and variance of *σ*_*B*_^2^ , which represents the between-cluster variance. *β*_conditional_ denotes the conditional regression coefficients. Model parameters can be estimated using maximum likelihood [15].

Both GEE and RELR are commonly used statistical analysis methods for analyzing binary outcomes in CRTs [1]; however, the two methods do not estimate the same parameter. As described above, the GEE method allows one to estimate the marginal or PA intervention effect, whereas RELR allows one to estimate the conditional or CS intervention effect [9,16,17]. Neuhaus has suggested that marginal models are preferable for testing the effects of cluster-level covariates [17]. In cluster randomization trials, the intervention is a cluster-level exposure variable and, thus, GEE approach may be preferable to RELR. Nevertheless, RELR may remain relevant for the analysis of CRTs since Neuhaus has demonstrated that for a binary outcome, marginal treatment effect tends to be smaller than conditional treatment effect: *β*_*m* arg *inal*_ = *β*_*conditinal*_(1 − *ρ*), where ρ is the intracluster correlation coefficient. In addition, different assumptions are required for the two models regarding missing data. The marginal model using GEE method requires data to be missing completely at random, whereas the cluster-specific model using RELR requires data to be missing at random. Both GEE and RELR are valid for analyzing binary outcomes in CRTs under the assumption of CDM.

### Missing data strategies

In this paper, we consider three strategies to handle missing binary outcomes in CRTs: 1) complete case analysis; 2) standard MI using logistic regression; and 3) within-cluster MI using logistic regression. The performance of GEE method and RELR is compared after missing data are handled by the above strategies.

Complete case analysis has been an attractive method to handle the missing data due to its simplicity. In adopting this strategy, only subjects with complete data are included for analysis, while subjects with missing data are excluded.

MI is widely applied to missing data problems. Rubin [18] described MI as a three-step process: 1) replace each missing value with a set of plausible values that represent the uncertainty about the right value to impute; 2) analyze the multiple imputed datasets independently using complete-data methods; and 3) combine the results from the multiple analyses, which allows the uncertainty regarding the imputation to be taken into account.

The standard MI using logistic regression method is now described in detail. The Within-cluster MI strategy is consists of applying the standard MI method to impute missing data for each cluster independently.

Standard multiple imputation using logistic regression is implemented through the following steps:

First, fit a logistic regression using the observed outcome and covariates to obtain the posterior predictive distribution of the parameters:

(3) $$\text{logit}(\text{Pr}\left(y_{\text{obs}}=1)\right)=\beta_{0}+\beta_{1}x_{1}+\cdots +\beta_{k}x_{k}\text{,}$$

where *y*_*obs*_ is the observed binary outcome of a subject, *x*_*i*_, *i* = 1, …, *k*, denotes the *i*^*th*^ individual or cluster-level covariate of the corresponding subject (two covariates are included in this study: treatment group and the variable associated with the missingness), *β* = (*β*_0_, *β*_1_, …, *β*_*k*_) denotes the regression coefficients. The regression parameter estimates $\hat{\beta }=\left({\hat{\beta }}_{0},{\hat{\beta }}_{1},\ldots ,{\hat{\beta }}_{k}\right)$ and the associated covariance matrix *V* are obtained to construct the posterior distribution of the parameters.

Second, draw new parameters $\tilde{\beta }=\left({\tilde{\beta }}_{0,}{\tilde{\beta }}_{1},\ldots ,{\tilde{\beta }}_{k}\right)$ from the posterior distribution, where $\tilde{\beta }=\hat{\beta }+V_{h}^{\prime }Z,V_{h}^{\prime }$ is the upper triangular matrix in the Cholesky decomposition, *V* = *V*_*h*_^′^*V*_*h*_, and *Z* is a vector of *k*+1 independent random Normal variates.

Third, for each subject with a missing outcome *y*_*mis*_ and observed covariates *x*_1_, …, *x*_*k*_, compute $p=\frac{\text{exp}\left({\tilde{\beta }}_{0}+{\tilde{\beta }}_{1}x_{1}\cdots +{\tilde{\beta }}_{k}x_{k}\right)}{1+\text{exp}\left({\tilde{\beta }}_{0}+{\tilde{\beta }}_{1}x_{1}\cdots +{\tilde{\beta }}_{k}x_{k}\right)}$ as the expected probability of *y*_*mis*_ = 1.

Fourth, draw a random Uniform variate *u*, 0 ≤ *u* ≤ 1. If *u* < *p*, then impute *y*_*mis*_ = 1, otherwise, impute *y*_*mis*_ = 0.

The above steps imply two assumptions: first, subjects are independent, which essentially ignores the similarity of subjects from the same cluster and; second, the missing data are imputed based on the PA treatment effect.

### Combination of results from different imputed data sets

Suppose *M* sets of imputed values are generated. *M* estimates of the treatment effects *β*^(1)^, *β*^(2)^, …, and*β*^(*M*)^ with corresponding variance estimates *V*^(1)^, *V*^(2)^, …, and *V*^(*M*)^ are obtained after GEE or RELR are applied to the multiple imputed datasets. The pooled treatment effect estimate from MI is calculated as $\bar{\beta }=\frac{1}{M}\sum_{m=1}^{M}\beta^{\left(m\right)}$. Its variance estimate is calculated as $V=W+\left(1+\frac{1}{M}\right)B$, where $W=\frac{1}{M}\sum_{m=1}^{M}V^{\left(m\right)}$ is the average within-imputation variance, and $B=\frac{1}{M-1}\sum_{m=1}^{M}{\left(\beta^{\left(m\right)}-\bar{\beta }\right)}^{2}$ is the between-imputation variance. As recommended by Barnard and Rubin [3,19], the adjusted degree of freedom is calculated for CRTs as $v_{\text{adj}}={\left(\frac{1}{v_{M}}+\frac{V}{W}\frac{v_{\text{com}}+3}{v_{\text{com}}+1}\frac{1}{v_{\text{com}}}\right)}^{-1}$ , where $v_{M}=\left(M-1\right){\left(1+\frac{M}{M+1}\frac{W}{B}\right)}^{2}$ is the degree of freedom when subjects are assumed to be independent, and *v*_*com*_ is the degree of freedom for the complete data test; for example, if there are *k* (*k*>2) clusters in each of the two study groups, *v*_*com*_ = 2(*k* − 1).

### Simulation study

**Figure 1:**
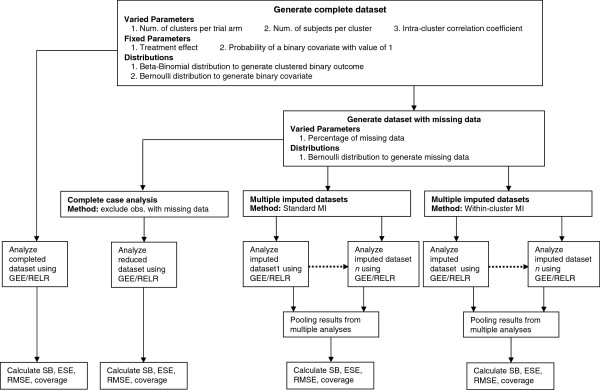
**Schematic overview of the simulation study.** Abbreviations: MI, multiple imputation; GEE, generalized estimating equations; RELR, random-effects logistic regression; SB, standardized bias; ESE, empirical standard error; RMSE, root mean square error; obs., observations.

The schematic overview of the simulation study is illustrated in Figure 1. This simulation study is implemented in SAS 9.2 (Cary, NC). The *mi* procedure is used to implement the MI, *genmod* and *nlmixed* procedures are used to estimate the intervention effect and its standard error from GEE approach and RELR respectively, and the *mianalyze* procedure is used to obtain the pooled estimate and its standard error across multiple imputed datasets. For *nlmixed* procedure, maximum likelihood estimation via adaptive Gaussian quadrature and a dual quasi-Newton optimization algorithm are used.

According to the review of CRTs in primary care by Eldrige *et al.*[20], CRTs can be categorized into two types: S-design and L-design, which refer to the design settings of CRTs with a small and a large number of clusters per arm, respectively. Design parameters for CRTs in this simulation study are guided by the empirical findings that larger values of intracluster correlation coefficient tend to be associated with studies having a small number of participants within each cluster [21]. The choices of these parameters are:

(1) For CRTs with 5 clusters per arm (S-design) and 500 subjects per cluster, ICC was set to be 0.001, 0.01 or 0.05.

(2) For CRTs with 20 clusters per arm (L-design) and 50 subjects per cluster, ICC was set to be 0.01, 0.05, or 0.1.

(3) For CRTs with 30 clusters per arm (L-design) and 30 subjects per cluster, ICC was set to be 0.05, 0.1, or 0.2.

Only two-arm, balanced, and completely randomized CRTs are considered in this study. The clustered binomial responses are generated using a beta-binomial distribution [22]. The prevalence of outcome for intervention and control arms is assumed to be 30% and 40% respectively. In addition, another binary covariate is generated, which has an equal chance of taking the value of 0 or 1 and is independent of the intervention and the outcome. For any percentage of missing data, we consider that subjects with value of 1 for this binary covariate are 1.3 times more likely to have missing outcome than subjects with a value of 0 for this covariate. For each combination of design parameters, we generate 1000 replications to achieve enough precision for estimating treatment effect [23]. Choices of the percentage of missing binary outcome are 0% (complete data), 15%, and 30%. We generate 5 replacements for each of the missing data.

Four quantities are chosen to evaluate the performance of GEE method and RELR: 1) standardized bias calculated as $\left|\frac{\text{Average}\;\text{of}\;\text{estimates}-\text{parameter}}{\text{standard}\;\text{deviation}\;\text{of}\;\text{estimates}}\right|$; 2) root mean squared error (RMSE) defined as $\sqrt{E_{\beta }\left[{\left(\hat{\beta }-\beta \right)}^{2}\right]}$, where $\hat{\beta }$ and *β* are the estimated treatment effect and its true value respectively; 3) coverage probability, which is the proportion of times that the nominal 95% confidence interval contains the true treatment effect across all simulation replications; and 4) empirical standard error of the treatment effect calculated as the average of standard errors of the estimated treatment effects across all simulation replications.

## Results

### Empirical standard error

**Figure 2:**
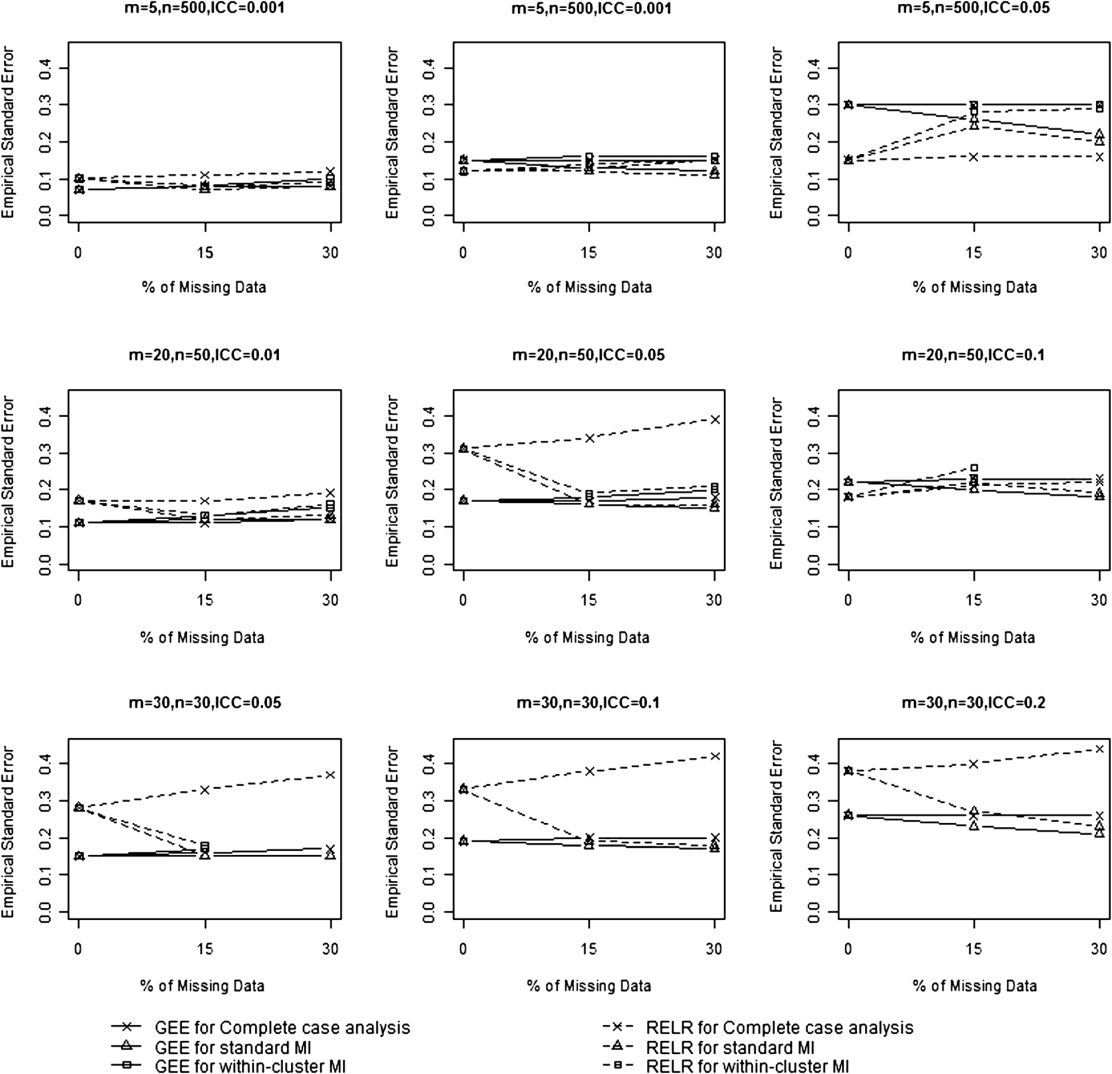
Comparison of empirical standard error.

The empirical standard errors from GEE method and RELR for different design scenarios are presented in Table 1 and Figure 2. When complete case analysis was used to handle missing data, empirical standard errors from GEE and RELR for all designs of CRTs increased with the increasing percentage of missing data. The magnitude of increase for the GEE method depended on the VIF of CRTs: the larger the VIF, the smaller amount of increase. In contrast, the magnitude of increase for the RELR depended on the cluster size: the smaller the cluster size, the larger amount of increase.

**Table 1:** Comparison of empirical standard error

**Design of CRTs**			**VIF**^**4**^	**% of missing data**	**Complete case analysis**		**Standard MI**^**5**^		**Within-cluster MI**^**6**^	
**m**^**1**^	**n**^**2**^	**ρ**^**3**^			**GEE**^**7**^	**RELR**^**8**^	**GEE**	**RELR**	**GEE**	**RELR**
5 ^**9**^ (S-Design)	500			0%	0.07	0.10				
				15%	0.08	0.11	0.08	0.07	0.08	0.08
				30%	0.08	0.12	0.08	0.08	0.10	0.09
				0%	0.15	0.12				
				15%	0.15	0.13	0.13	0.12	0.16	0.14
				30%	0.15	0.15	0.12	0.11	0.16	0.15
				0%	0.30	0.15				
				15%	0.30	0.16	0.26	0.24	0.30	0.28
				30%	0.30	0.16	0.22	0.20	0.30	0.29
20 (L-Design)	50			0%	0.11	0.17				
				15%	0.11	0.17	0.12	0.12	0.13	0.13
				30%	0.12	0.19	0.12	0.13	0.15	0.16
				0%	0.17	0.31				
				15%	0.17	0.34	0.16	0.16	0.18	0.19
				30%	0.18	0.39	0.15	0.16	0.20	0.21
				0%	0.22	0.18				
				15%	0.23	0.21	0.20	0.22	0.23	0.26
				30%	0.23	0.22	0.18	0.19	NA	NA
30 (L-Design)	30			0%	0.15	0.28				
				15%	0.16	0.33	0.15	0.15	0.17	0.18
				30%	0.17	0.37	0.15	0.15	NA	NA
				0%	0.19	0.33				
				15%	0.20	0.38	0.18	0.19	NA	NA
				30%	0.20	0.42	0.17	0.18	NA	NA
				0%	0.26	0.38				
				15%	0.26	0.40	0.23	0.27	NA	NA
				30%	0.26	0.44	0.21	0.23	NA	NA

Empirical standard error is defined as the average of standard errors of the estimated treatment effects across all simulation replications. The empirical standard errors obtained when 0% data are missing are considered as references for comparing with those obtained when 15% or 30% data are missing.

Note: 1. m: Number of clusters per trial arm. 2. n: Number of subjects per cluster.

3. ρ: intracluster correlation coefficient; 4. VIF: Variance inflation factor, i.e. 1+(m-1)ρ; 5. Standard MI: Standard multiple imputation using logistic regression method.

6. Within-cluster MI: Within-cluster multiple imputation using logistic regression method, which is not applicable (NA) for some L-design of cluster randomized trials.

7. GEE: Generalized estimating equations. 8. RELR: Random-effects logistic regression.

9. For CRTs with 5 clusters per arm, modified standard errors are provided.

When standard MI was used to impute missing data, empirical standard errors from the GEE method were acceptable for CRTs with VIF<3 in terms of yielding similar or slightly larger empirical standard errors compared to those obtained from analyzing the complete data. However, they were underestimated for CRTs with VIF≥3. This is because standard MI strategy assumes data are independent, and cluster effect may be safely ignored for CRTs with VIF<3 when imputing missing data. In contrast, empirical standard errors from RELR were not similar as those obtained from analyzing complete data. This is because that the imputed datasets were obtained based on the estimated PA treatment effect and corresponding underestimated standard error, which led to a difference between the standard error estimated from RELR based on the imputed datasets and that based on the complete data.

Within-cluster MI was not applicable for L-design of CRTs, which usually had a small cluster size, since all outcomes in a cluster were missing or all observed outcomes had identical values, which caused the imputation procedure to fail. In the cases when within-cluster MI was applicable and used to impute the missing data, empirical standard errors from GEE method were acceptable for CRTs with VIF≥3; however, for CRTs with VIF<3, empirical standard errors were inflated. This is because when within-cluster MI was used to impute the missing data, the clustering effects were accounted for by imputing missing data based on the observed information within the same cluster as the missing data, therefore, the empirical standard errors for GEE were acceptable for CRTs with VIF≥3. The empirical standard errors from RELR were acceptable only when the cluster size is large (>50) and the ICC is small (≤0.01).

### Standardized bias

**Figure 3:**
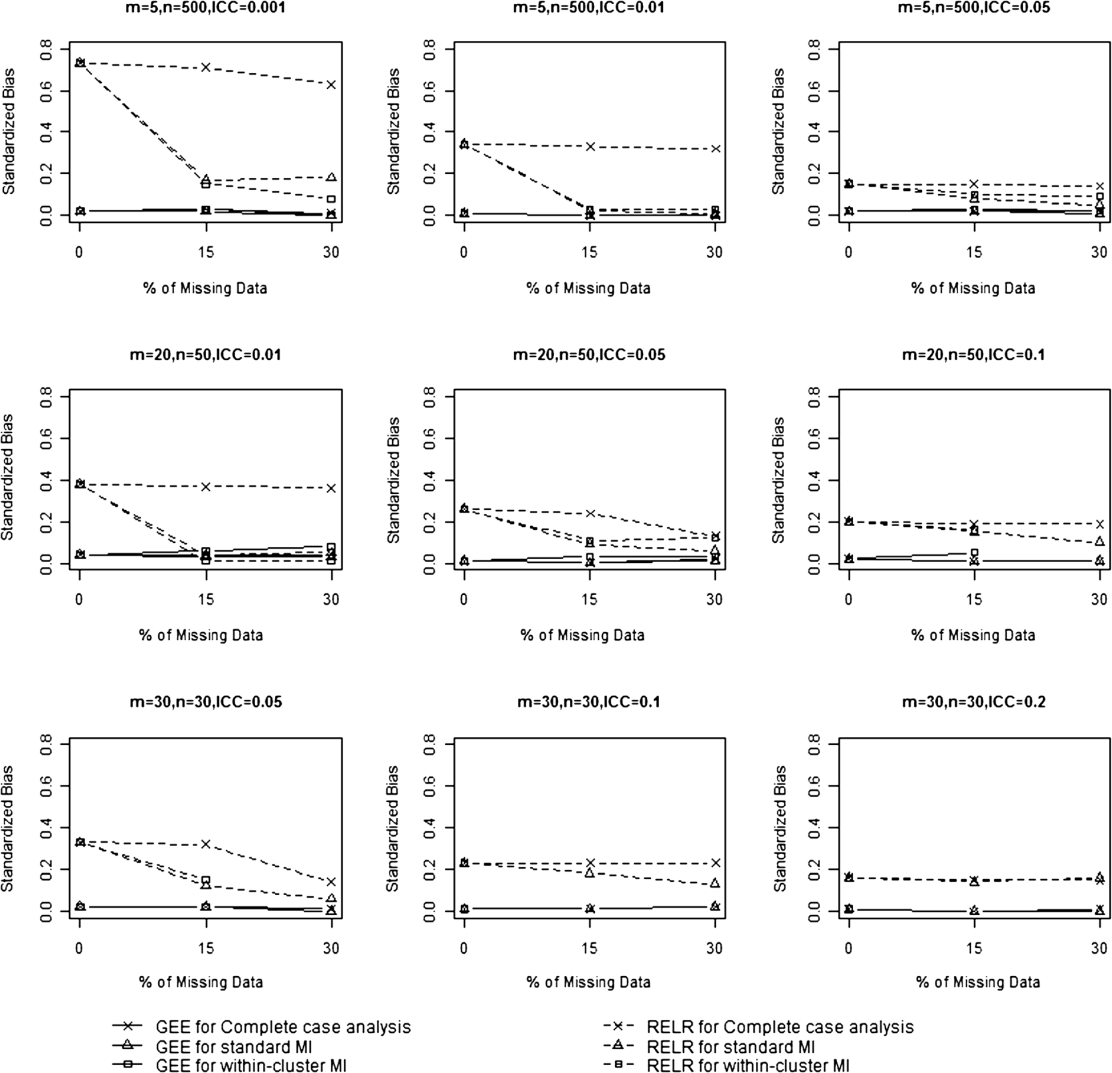
Comparison of standardized bias.

The standardized biases from GEE method and RELR for different design scenarios are presented in Table 2 and Figure 3. Standardized biases from GEE method were close to zero for any design settings and percentage of missing data, no matter which missing data strategy was used. In contrast, standardized biases for RELR were relatively larger. When complete case analysis was used to handle missing data, standardized biases for RELR did not change substantially with increasing percentage of missing data for S-design with a large design effect (VIF>3) and L-design with a large ICC (ICC≥0.1); however, standardized biases changed largely with an increasing percentage of missing data for other scenarios. When missing data were imputed by standard MI or within-cluster MI prior to statistical analysis, standardized biases for RELR were much smaller than those obtained by analyzing complete data (i.e. 0% missing data) using the same statistical method.

**Table 2:** Comparison of standardized bias

**Design of CRTs**			**VIF**^**4**^	**% of missing data**	**Complete case analysis**		**Standard MI**^**5**^		**Within-cluster MI**^**6**^	
**m**^**1**^	**n**^**2**^	**ρ**^**3**^			**GEE**^**7**^	**RELR**^**8**^	**GEE**	**RELR**	**GEE**	**RELR**
5 ^**9**^ (S-Design)	500			0%	0.02	0.73				
				15%	0.03	0.71	0.02	0.17	0.03	0.15
				30%	0.01	0.63	0.00	0.18	0.00	0.08
				0%	0.01	0.34				
				15%	0.00	0.33	0.00	0.02	0.00	0.03
				30%	0.00	0.32	0.00	0.01	0.00	0.03
				0%	0.02	0.15				
				15%	0.02	0.15	0.02	0.08	0.03	0.10
				30%	0.02	0.14	0.01	0.05	0.02	0.09
20 (L-Design)	50			0%	0.04	0.38				
				15%	0.04	0.37	0.03	0.04	0.06	0.01
				30%	0.04	0.36	0.03	0.05	0.08	0.01
				0%	0.01	0.26				
				15%	0.00	0.24	0.00	0.09	0.03	0.11
				30%	0.02	0.13	0.01	0.06	0.03	0.12
				0%	0.02	0.20				
				15%	0.01	0.19	0.01	0.15	0.05	0.16
				30%	0.01	0.19	0.01	0.10	NA	NA
30 (L-Design)	30			0%	0.02	0.33				
				15%	0.02	0.32	0.02	0.12	0.02	0.15
				30%	0.01	0.14	0.00	0.06	NA	NA
				0%	0.01	0.23				
				15%	0.01	0.23	0.01	0.18	NA	NA
				30%	0.02	0.23	0.02	0.13	NA	NA
				0%	0.01	0.16				
				15%	0.00	0.15	0.00	0.14	NA	NA
				30%	0.01	0.15	0.00	0.16	NA	NA

Standardized bias is defined as the difference between the expectation of the estimator and the parameter, divided by the standard deviation of the estimator. Standardized biases obtained when 0% data are missing are considered as references for comparing with those obtained when 15% or 30% data are missing.

Note: 1. m: Number of clusters per trial arm. 2. n: Number of subjects per cluster.

3. ρ: Intracluster correlation coefficient; 4. VIF: Variance inflation factor, i.e. 1+(m-1)ρ; 5. Standard MI: Standard multiple imputation using logistic regression method.

6. Within-cluster MI: Within-cluster multiple imputation using logistic regression method, which is not applicable (NA) for some L-design of cluster randomized trials.

7. GEE: Generalized estimating equations. 8. RELR: Random-effects logistic regression.

9. For CRTs with 5 clusters per arm, modified standard errors are provided.

The magnitude of standardized bias was dependent on the original data structure, i.e. how the data were generated, how the missing data were handled, and which statistical model was used for analysis. As described in the previous section, the clustered binary data were generated using a beta-binomial distribution, which assumed a PA treatment effect. Since complete case analysis did not change the original data structure under the assumption of CDM, the PA and CS treatment effects estimated from the GEE and RELR were quite consistent with those estimated based on complete data (i.e. datasets without missing values). The relationship between the PA and the CS treatment effects estimated from GEE method and RELR respectively still held; however, when either standard MI or within-cluster MI was used, the imputed values were obtained based on the estimated PA treatment effect and corresponding underestimated standard error, which largely distorted the CS treatment effects estimated from RELR compared with those estimated based on complete data.

### Root mean squared error

**Figure 4:**
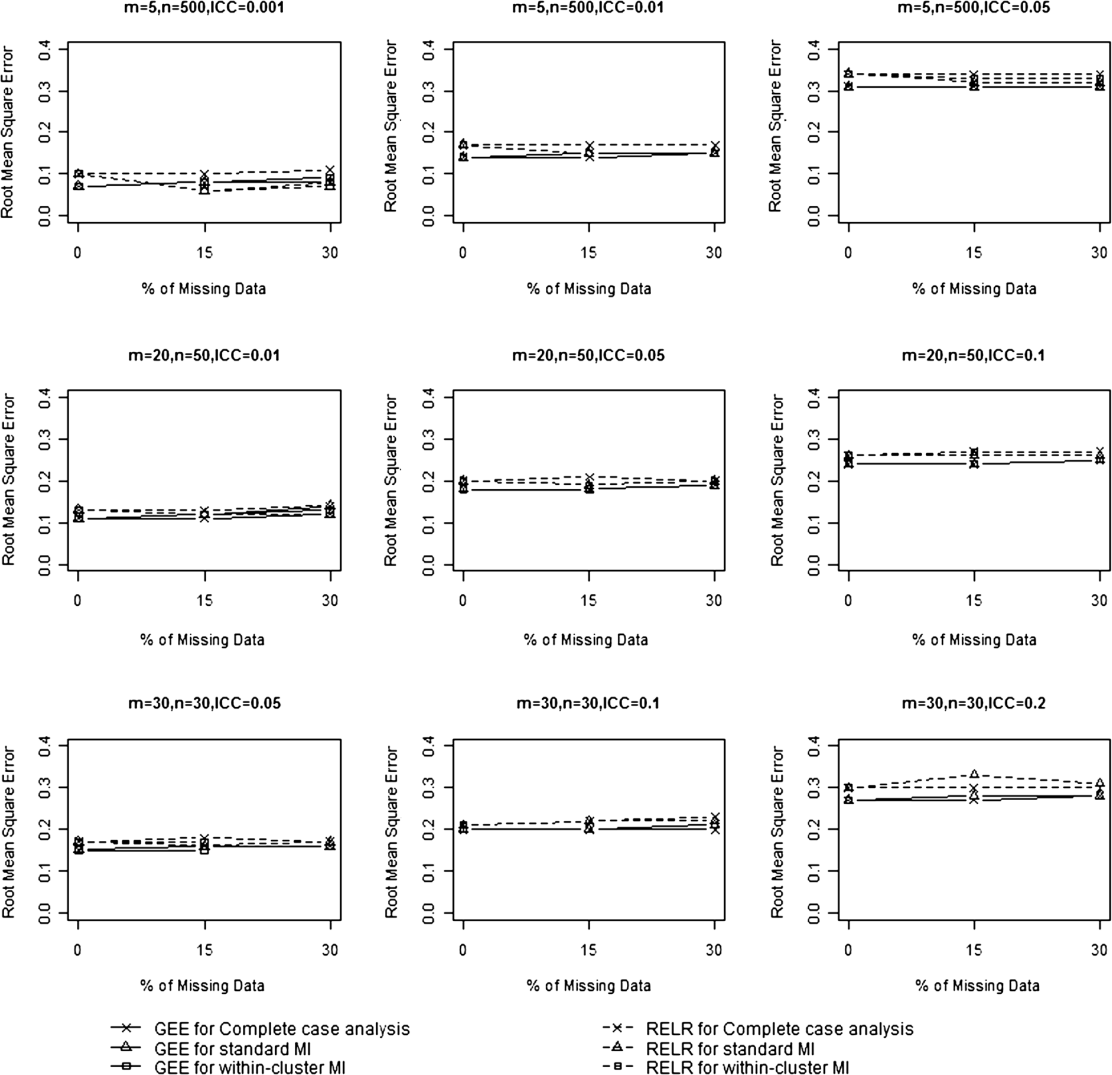
Comparison of root mean squared error.

The RMSE incorporates both the variance of the estimator and its bias, and measures the overall accuracy of the point estimator. RMSEs from GEE method and RELR for different design scenarios are presented in Table 3 and Figure 4. When complete case analysis was used to handle missing data, RMSEs from GEE method were very similar to those obtained based on complete data for all designs of CRTs with no larger than 15% missing data. With 30% missing values, the RMSEs from GEE were larger than those obtained based on analyzing complete data for the design of CRTs with a small design effect (VIF<3). Similarly, RMSEs from RELR were very similar to those obtained based on complete data for all designs of CRTs with no larger than 15% missing data; however, with 30% missing values, RMSEs from RELR were much larger than those obtained based on complete data for the design of CRTs with a small design effect (VIF<3) and a small cluster size (<50).

**Table 3:** Comparison of root mean squared error

**Design of CRTs**			**VIF**^**4**^	**% of missing data**	**Complete case analysis**		**Standard MI**^**5**^		**Within-cluster MI**^**6**^	
**m**^**1**^	**n**^**2**^	**ρ**^**3**^			**GEE**^**7**^	**RELR**^**8**^	**GEE**	**RELR**	**GEE**	**RELR**
5 ^**9**^ (S-Design)	500			0%	0.07	0.10				
				15%	0.08	0.10	0.08	0.06	0.08	0.06
				30%	0.08	0.11	0.08	0.07	0.09	0.08
				0%	0.14	0.17				
				15%	0.14	0.17	0.15	0.15	0.15	0.15
				30%	0.15	0.17	0.15	0.15	0.15	0.15
				0%	0.31	0.34				
				15%	0.31	0.34	0.31	0.32	0.31	0.33
				30%	0.31	0.34	0.31	0.32	0.31	0.33
20 (L-Design)	50			0%	0.11	0.13				
				15%	0.11	0.13	0.12	0.12	0.12	0.12
				30%	0.12	0.14	0.14	0.12	0.13	0.13
				0%	0.18	0.20				
				15%	0.18	0.21	0.18	0.19	0.18	0.19
				30%	0.19	0.20	0.19	0.20	0.19	0.20
				0%	0.24	0.26				
				15%	0.24	0.27	0.24	0.26	0.24	0.27
				30%	0.25	0.27	0.25	0.26	NA	NA
30 (L-Design)	30			0%	0.15	0.17				
				15%	0.16	0.18	0.16	0.16	0.15	0.17
				30%	0.16	0.17	0.16	0.17	NA	NA
				0%	0.20	0.21				
				15%	0.20	0.22	0.20	0.22	NA	NA
				30%	0.20	0.23	0.21	0.22	NA	NA
				0%	0.27	0.30				
				15%	0.27	0.30	0.28	0.33	NA	NA
				30%	0.28	0.30	0.28	0.31	NA	NA

Root mean squared error is defined as the square root of the mean squared error, which is the average squared difference between the estimated treatment effect and the true parameter. The root mean squared errors obtained when 0% data are missing are considered as references for comparing with those obtained when 15% or 30% data are missing.

Note: 1. m: Number of clusters per trial arm. 2. n: Number of subjects per cluster.

3. ρ: Intracluster correlation coefficient; 4. VIF: Variance inflation factor, i.e. 1+(m-1)ρ; 5. Standard MI: Standard multiple imputation using logistic regression method.

6. Within-cluster MI: Within-cluster multiple imputation using logistic regression method, which is not applicable (NA) for some L-design of cluster randomized trials.

7. GEE: Generalized estimating equations. 8. RELR: Random-effects logistic regression.

9. For CRTs with 5 clusters per arm, modified standard errors are provided.

When standard MI was used to impute missing data, RMSEs from GEE method increased with the percentage of missing data. With no larger than 15% missing data, the increase of RMSEs from GEE compared to those obtained based on complete data was not substantial. When the amount of missing values increased to 30%, RMSEs from the GEE method increased substantially for CRTs with a small design effect (VIF<3). In contrast, RMSEs from RELR method were much smaller than those obtained from analyzing complete data for most of the design scenarios. We should note that the small RMSE for RELR here was not an indication of more accurate or precise estimate for the treatment effect, but rather a result of biased CS treatment effects and the corresponding underestimated standard error.

When within-cluster MI was used to impute missing data, the same pattern for RMSEs from both GEE and RELR was observed as when standard MI was used to impute missing data.

### Coverage probability

**Figure 5:**
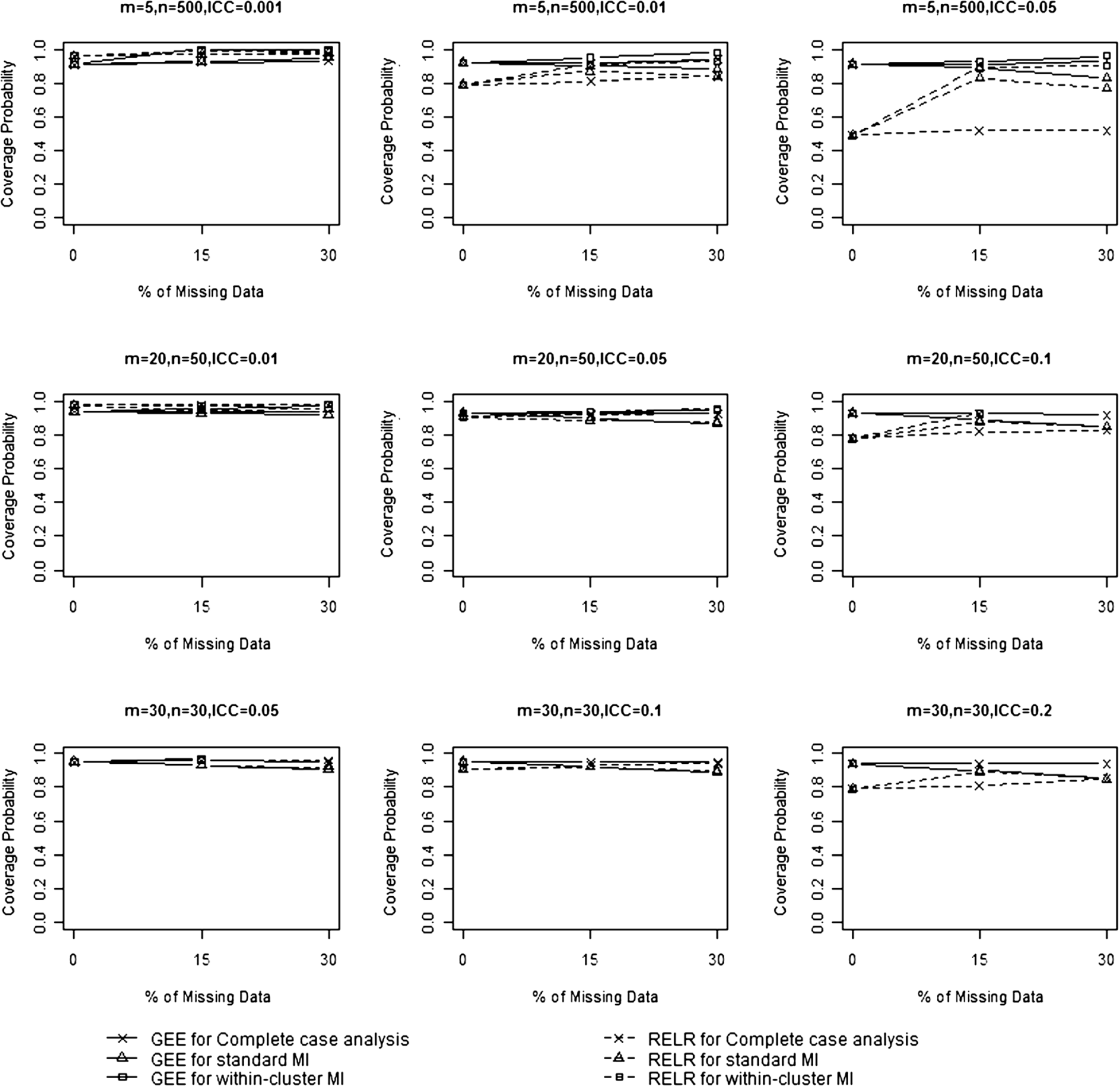
Comparison of coverage probability.

Table 4 and Figure 5 show the coverage probabilities from GEE method and RELR for different designs of CRTs. When complete case analysis was used to handle missing data, the coverage probabilities from GEE method were at least 0.90 for all the scenarios considered in this paper. The coverage probabilities from RELR were at least 0.95 for design of CRTs with a small design effect (VIF<3) but were very low for CRTs with a large design effect (VIF≥3).

**Table 4:** Comparison of coverage probability

**Design of CRTs**			**VIF**^**4**^	**% of missing data**	**Complete case analysis**		**Standard MI**^**5**^		**Within-cluster MI**^**6**^	
**m**^**1**^	**n**^**2**^	**ρ**^**3**^			**GEE**^**7**^	**RELR**^**8**^	**GEE**	**RELR**	**GEE**	**RELR**
5 ^**9**^ (S-Design)	500			0%	0.91	0.96				
				15%	0.92	0.97	0.93	0.97	1.00	0.99
				30%	0.93	0.97	0.95	0.98	1.00	0.99
				0%	0.92	0.79				
				15%	0.92	0.81	0.90	0.87	0.95	0.91
				30%	0.94	0.84	0.88	0.84	0.98	0.93
				0%	0.91	0.49				
				15%	0.91	0.52	0.89	0.83	0.93	0.89
				30%	0.93	0.52	0.83	0.77	0.96	0.90
20 (L-Design)	50			0%	0.94	0.98				
				15%	0.94	0.98	0.93	0.95	0.96	0.97
				30%	0.94	0.98	0.92	0.96	0.98	0.98
				0%	0.93	0.91				
				15%	0.93	0.92	0.90	0.89	0.94	0.94
				30%	0.93	0.93	0.87	0.88	0.95	0.96
				0%	0.93	0.78				
				15%	0.93	0.82	0.89	0.88	0.93	0.93
				30%	0.92	0.83	0.85	0.85	NA	NA
30 (L-Design)	30			0%	0.95	0.95				
				15%	0.96	0.96	0.93	0.93	0.97	0.96
				30%	0.95	0.96	0.91	0.92	NA	NA
				0%	0.95	0.91				
				15%	0.95	0.93	0.92	0.92	NA	NA
				30%	0.95	0.94	0.89	0.90	NA	NA
				0%	0.94	0.79				
				15%	0.94	0.81	0.90	0.89	NA	NA
				30%	0.94	0.85	0.85	0.85	NA	NA

Coverage probability is defined as the proportion of times that the nominal 95% confidence interval contains the true treatment effect across all simulation replications. Coverage probabilities obtained when 0% data are missing are considered as references for comparing with those obtained when 15% or 30% data are missing.

Note: 1. m: Number of clusters per trial arm. 2. n: Number of subjects per cluster.

3. ρ: Intra-cluster correlation coefficient; 4. VIF: Variance inflation factor, i.e. 1+(m-1)ρ; 5. Standard MI: Standard multiple imputation using logistic regression method.

6. Within-cluster MI: Within-cluster multiple imputation using logistic regression method, which is not applicable (NA) for some L-design of cluster randomized trials.

7. GEE: Generalized estimating equations. 8. RELR: Random-effects logistic regression.

9. For CRTs with 5 clusters per arm, modified standard errors are provided.

When standard MI was used to impute missing data, coverage probabilities from GEE method increased for CRTs with a small design effect but decreased for CRTs with a large design effect. Coverage probabilities from RELR increased for almost all designs of CRTs compared to those obtained by analyzing complete data using the same statistical analysis method. When within-cluster MI was used to impute missing data, the same pattern for the coverage probabilities from both GEE and RELR was observed as when standard MI was used to impute missing data. It should be noted that the higher coverage from RELR when either standard or within-cluster MI strategy was applied prior to the analysis was not an indication of high efficiency, but rather a result of biased CS treatment effects and the corresponding underestimated standard effort.

We noticed that the coverage probabilities from GEE were larger than the nominal level when within-cluster MI is applied prior to the analysis for CRTs with a small design effect and a large percentage of missing data. This is because within-cluster MI tends to provide larger standard errors of the estimated treatment effects (i.e. wider 95% confidence interval).

### Convergence problems

For the GEE method, at most 1 out of 1000 simulated datasets with S-design could not converge to a solution because they either encountered a non-positive definite matrix in the iterations or because there was no variation between the clusters in each arm. No convergence problems occurred for the simulated datasets based on the L-design. Lack of convergence was encountered more often for RELR than GEE. About 10 out of 1000 simulated datasets for some designs of CRTs could not converge for RELR due to negative estimates of between-cluster variance component during iteration.

## Discussion

In this paper, we compared the accuracy and efficiency of PA and CS models through a simulation study, in particular, the GEE method and the RELR respectively, for analyzing binary outcomes in CRTs with missing data. Results from the present simulation study show that under the assumption of CDM, the GEE method performs well as long as an appropriate strategy is applied to handle missing data based on the percentage of missing data and the design of CRTs. The appropriate strategy in this instance is using complete case analysis for any CRTs with a small percentage of missing outcomes (<15%), using standard MI to impute missing outcomes for CRTs with a small design effect (VIF<3), or within-cluster MI to impute missing outcomes for CRTs with a large design effect (VIF≥3) and cluster size (>50). In contrast, the RELR performs poorly when either standard or within-cluster MI strategy is used to impute missing data prior to the analysis.

Results from the present comprehensive simulation study also imply that MI using random-effects logistic regression may not appropriate for imputing binary outcomes in CRTs. This is because that if the underlying data structure assumes a PA treatment effect, the MI using random-effects logistic regression, which impute missing data based on the CS treatment effect, may distort the original data structure and lead to invalid inference. Moreover, the convergence problems will greatly hinder the application of this method for imputing missing binary data. This implication seems to be in contradiction with current literature: for example, Taljaard *et al.*[24] proposed mixed-effects regression imputation strategies to handle missing continuous outcomes in CRTs. Results from that study showed that the mixed-effects regression imputation strategy takes into account the between-cluster variance and therefore provides valid inferences for the treatment effect. In a previous study [11], we proposed MI using random-effects logistic regression to impute missing binary outcomes in CRTs and found that this strategy may be valid for imputing binary outcomes in CRTs. These two studies reached a different conclusion from the present simulation study since the mixed-effects regression imputation strategy by Taljaard *et al.* is used to handle the missing continuous outcome, and the MI using random-effects logistic regression by Ma *et al.* is based on a real dataset which has relatively large ICC, number of clusters per arm, and number of subjects per cluster, which limited the generalizability of their conclusions to more general settings.

MI has been accepted as a solution for missing data problems in many settings. Both GEE and RELR are commonly used for analyzing binary data in CRTs [1]. Results from this paper also imply that the choice of statistical analysis method and imputation method should reflect the same data structure as the inherent structure of the original data; otherwise, valid or improved inferences will not be achieved. For researchers with thorough understandings of the GEE method, RELR, CRTs, and the MI, results from this present study may not entirely surprising; however, the application of imputation and analysis methods in practice for CRTs does not reflect this finding. Some CRTs used mixed effects models for statistical analysis, but fixed-effects for clusters in imputation [25-28]. In some other CRTs [29-31], no details were provided on which imputation procedure was applied. Findings from this simulation study urge caution on the use of RELR in the analysis of data from CRTs when missing binary outcomes are imputed by either standard or within-cluster MI strategy, thus improve the statistical practice in epidemiological research.

There are certain limitations to the current study. First, performance of the marginal model and cluster-specific model was assessed only for CRTs with a completely randomized design. Other designs such as the matched pairs design and stratified randomized design are also used for CRTs but were not considered in this study. Second, only CRTs with balanced design were considered; however, settings found more often in empirical situations, such as unequal numbers of subjects per cluster, or unequal number of clusters in each trial arm, were not considered in this study. These design restrictions were made to understand the performance of the methods in simple scenarios. Further research is required to assess whether our findings are relevant to more general settings. Third, there are two main approaches in handling missing data: likelihood based analyses and imputation [3]. In this paper, only complete case analysis, standard and within-cluster MI using logistic regression method to handle the missing data were considered; therefore, the conclusion from this paper regarding to the performance of RELR may not be applicable when missing data are handled using likelihood based analyses or other imputation methods. Further research may investigate the scenarios when missing data are handled by likelihood based analysis. Fourth, MI strategies investigated in this project were implemented using SAS. Other software packages, such as STATA and MLwiN, also provide MI procedures or macros. We think that similar results may be expected if the procedure in STATA is used since it generates imputed values from a prediction model using logistic regression — which is similar to the MI procedure for imputing missing binary data in SAS; while different results may be expected if the macros in MLwiN is used — because it generates imputed values using the random-effects logistic regression model. Finally, results from GEE and RELR are different since the two models estimate different parameters as outlined in the previous section. The intervention effect in the simulation has a population-average interpretation since the beta-binomial model is used to specify an overall unconditional probability within each trial arm, which gives preference to the GEE model.

## Conclusions

Under the assumption of CDM, GEE method performs well as long as an appropriate missing data strategy is adopted based on the design of CRTs and the percentage of missing data. In contrast, RELR dose not perform well when either standard or within-cluster MI strategy is applied to impute missing data prior to the analysis.

## Abbreviation

CRTs: Cluster randomized trials; ICC: Intracluster correlation coefficient; VIF: Variance inflation factor; PA: Population-averaged; CS: Cluster-specific; GEE: Generalized estimating equations; RELR: Random-effects logistic regression; RMSE: Root mean squared error; CDM: Covariate dependent missingness.

## Competing interests

The authors declare that they have no competing interests.

## Authors’ contributions

JM, PR, JB, and LT conceived the research question. JM conducted literature review, designed and implemented the simulation study, composed the initial draft of the manuscript, and revised the manuscript. LT oversaw the design and implementation of the study, revised the manuscript. PR and JB provided assistance with design of the simulation study. All authors read and approved the final manuscript.

## Pre-publication history

The pre-publication history for this paper can be accessed here:

http://www.biomedcentral.com/1471-2288/13/9/prepub
